# Influence of Abrasive Treatment on a Transformation of Zirconium Oxide Used in Dental Prosthetics

**DOI:** 10.3390/ma15124245

**Published:** 2022-06-15

**Authors:** Kinga Regulska, Bartłomiej Januszewicz, Leszek Klimek

**Affiliations:** Institute of Materials Science and Engineering, Faculty of Mechanical Engineering, Lodz University of Technology, 1/15 Stefanowskiego Str., 90-924 Lodz, Poland; bartlomiej.januszewicz@p.lodz.pl (B.J.); leszek.klimek@p.lodz.pl (L.K.)

**Keywords:** zirconium oxide (ZrO_2_), ceramics, crystallographic structure, XRD, phase transformation

## Abstract

Zirconium oxide is a ceramic most often used in the field of dentistry for permanently cementing the substructures of prosthetic restorations in patients. The surface of zirconium oxide should be prepared properly because in the next stage it must be covered with porcelain. The success of prosthetics treatment depends on various factors, but it has been reported that the transformation of zirconium oxide plays a key role. The purpose of the research was to investigate the effect of abrasive blasting on the transformation of zirconium oxide. The research has shown that this type of surface treatment causes the transformation of the tetragonal phase into a monoclinic one. The samples were examined using X-ray diffraction (XRD). The study confirmed the assumption.

## 1. Introduction

Nowadays, the awareness of patients and their expectations regarding visits to dental offices are both increasing, and the aesthetic dentistry department is becoming increasingly popular. An increasing amount of patients choose restoration, which can improve their appearance by providing a beautiful, bright, and healthy smile.

Zirconium oxide (ZrO_2_) is an oxide ceramic. It is characterized by its very good strength properties. Its tensile strength ranges from 900–1200 MPa, its compressive strength 2000 MPa, and its cracking strength 4–6 MPa m1/2 [1,2]. It is also a highly durable material. ZrO_2_ restorations can carry loads of 750 N. In a pure form, the material has a milky-white color and is translucent to some extent [1]. Translucency is one of the main reasons why the patients choose a restoration based on zirconium. The light passes through the zirconium and thanks to this presence of artificial teeth is not visible. In comparison with metal substructures, light cannot pass through, and in dark places with lamp lights only a “black hole” effect can occur. Restorations from ZrO_2_ are much more natural and aesthetic than others. All of these factors make ZrO_2_ the material of the future.

ZrO_2_ occurs in three structural forms: cubic phase, a monoclinic form, and a tetragonal form. Crystals have a form of fine grains with 0.2–0.5 μm size [1]. In prosthetics, the tetragonal form is used. This form is unstable at ambient temperature and because of this zirconium oxide is stabilized by adding other oxides to their structure (such as yttrium oxide and magnesium oxide) [3,4].

One of the biggest advantages of this material is its perfect biocompatibility with oral tissues. Prosthetic restorations with a cup from zirconium oxide are produced using the CAD/CAM method which makes it possible to achieve a marginal tightness on the order of 30 μm, which is over three times better than the generally permissible value (100 μm). The whole process is automatic, and the cups are produced from ready-made blocks of zirconium oxide by using a milling machine. This allows for the elimination of the disadvantages that arise from the traditional casting of metal alloys [5,6,7]. 

In dental prosthetics, zirconium oxide has mainly been used in the manufacturing of prosthetic cups for crowns and implant restorations. Due to its opaque structure, it easily covers the ground tooth pillar, and the ZrO_2_ itself can be easily covered with veneering material due to its light color. In addition, often before sintering the cup, staining methods are used to obtain a color of the cup that is as close as possible to the color of the abutment tooth [6,8].

The replacement of a metal cup with other materials is a procedure that is used increasingly often. Zirconium oxide has emerged as one of the most promising alternatives. It is characterized with excellent aesthetic value because it is a material with a light color and it is also easy to cover with veneering porcelain. ZrO_2_ is a biocompatible material [4,9,10,11]. In addition, clinical studies did not reveal the occurrence of allergies in people with prosthetic restorations on a zirconium oxide substructure, which is the opposite of those restorations on metal, where a large percentage of owners of such restorations have, for example, a bluish discoloration of the gingiva, commonly known as the “gingival margin” [12,13,14].

A factor of great importance for improving the quality of the connection between zirconium oxide and veneering ceramics is increasing its adhesion to the substrate by increasing the roughness. Since the ceramics are applied in the form of an aqueous suspension, they influence the cavities formed during processing, which enables a mechanical connection with the substrate [6,14,15]. In order to increase the retention before applying the veneering ceramics, zirconium oxide frameworks are therefore subjected to various processes, such as grinding, sandblasting, polishing, and laser ablation [16,17]. Sometimes chemical etching is used [18]. These processes are less invasive, but not satisfying, and that is why machining is necessary. It should also be considered that the surface treatments have different effects on the liquid ceramic wettability of the materials used for the substructures [19,20], which facilitate its flow and improves retention. The surface treatment of the material plays a key role and has a direct impact on the success of the prosthetic restoration and the duration of its use. A poorly prepared material surface results in defects in the internal structure of the zirconium oxide, which may lead to damage to the ceramics in the form of scratches, cracks, material chips, or in the worst case—fractures. All this means that the supplement may become useless, and it will be necessary to replace it with a new one [21,22]. Some of the examinations prove that despite the use of stabilizers in a structure of zirconium oxide, during the surface treatment processes, changes occur in its internal structure and the tetragonal phase turns into a monoclinic one [6].

Mechanical working (sandblasting and griding) can cause the inflow of supercritical energy, which in turn results in the formation of surface distortions in the crystal structure of the material and leads to the phase transformation of ZrO_2_ [23]. Studies have shown that abrasive blasting is not a neutral process for the zirconium oxide structure, as it results in erosive damage in the form of scratches, microcracks, fissures, or tearing out the material grains from its surface [24]. On the other hand, according to other studies after sandblasting, a significant amount of embedded abrasive may remain in the surface of the substrate. The surface quality is dependent on the grain hardness and the processing parameter-pressure, as well as the type and size of the grain. However, this is a very important stage of the material treatment because it is caused by increasing the roughness and thus improving the connection between ZrO_2_ and the veneering ceramics [25,26,27,28]. Hallmann and other researchers have shown that the abrasive particles not only adhere to the substrate of the zirconium oxide restoration, but also interact with it, leading to the formation of covalent bonds between the abrasive particles and the ceramic surface [29].

The biggest challenge is to find a surface treatment which helps to reduce or minimalize a phase transformation—one which helps to improve the quality of prosthetic restorations and increases their lifetime.

The phase changes in zirconium oxide caused by abrasive blasting can have a negative effect on the behavior of the restoration. It is important to carry out the treatment with such parameters (pressure, size, and type of the abrasive grain) that will cause as little transformation as possible. Therefore, the aim of the study is to investigate the effect of these abrasive blasting parameters on the degree of transformation from the tetragonal phase into monoclinic phase.

## 2. Materials and Methods

The materials used for testing were cylindrical zirconium oxide samples 3Y-TZP CeramillZi (Shenzhen Upcera Dental Technology Co., Ltd.; High-Tech Industry Park, Nanshan District, Shenzhen, Guangdong, China), which after being cut from the block were sintered in a furnace (CeramillTherm; AmannGirrbach AG, Koblach, Austria) using a universal program (8°/min from 200 °C to 1450 °C, 2 h at a constant temperature of 1450 °C). The entire sintering process took about 10 h. The shrinkage of the material was approximately 21%. After sintering, the specimens were 20 mm in diameter and 10 mm in height. The composition of the samples is given in Table 1.

After the sintering process, the samples were divided into groups, and within each group the surfaces of the samples were subjected to the following treatments:

A—sandblasting, abrasive—Al_2_O_3_ 60 µm, pressure 200 kPa;
B—sandblasting, abrasive—Al_2_O_3_ 60 µm, pressure 400 kPa;
C—sandblasting, abrasive—Al_2_O_3_ 110 µm, pressure 200 kPa;
D—sandblasting, abrasive—Al_2_O_3_ 110 µm, pressure 400 kPa;
E—sandblasting abrasive—Al_2_O_3_ 250 µm, pressure 200 kPa;
F—sandblasting, abrasive—Al_2_O_3_ 250 µm, pressure 400 kPa;
G—sandblasting, abrasive—SiC, 60 µm, pressure 200 kPa;
H—sandblasting, abrasive—SiC, 60 µm, pressure 400 kPa;
I—sandblasting, abrasive—SiC, 110 µm, pressure 200 kPa;
J—sandblasting abrasive—SiC, 110 µm, pressure 400 kPa;
K—sandblasting, abrasive—SiC, 250 µm, pressure 200 kPa;
L—sandblasting, abrasive—SiC, 250 µm, pressure 400 kPa.

An initial sample was used as a reference sample, which was used after milling without surface treatment. The phase contents calculated for it were presented in the previously published work [29]. In this sample, only the presence of the tetragonal phase was found.

**Figure 1:**
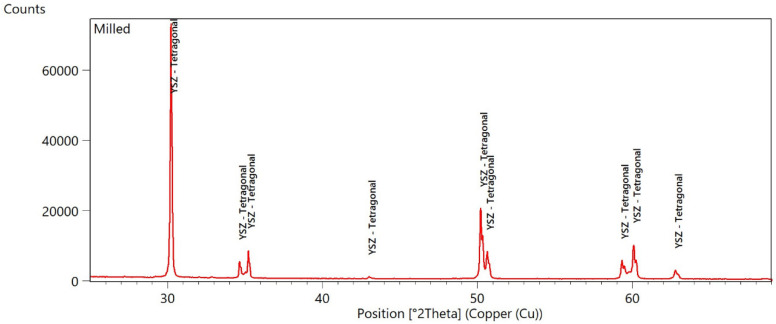
Milled and sintered sample.

Diffraction pattern for untreated sample is presented in Figure 1.

The samples prepared in this way were subjected to the following qualitative and quantitative diffractometric tests which aimed at determining the phases occurring in individual samples and calculating their content.

Diffractometric experiments were performed on the PANalytical Empyrean X-ray diffractometer (PANalytical, Almelo, The Netherlands). The diameter of the goniometer was 240 mm, and the device operated in the Bragg-Brentano geometry in the θ-θ system or the geometry of a constant angle of incidence. The primary beam was obtained by means of an X-ray tube with a copper (Cu) anode emitting characteristic radiation with a wavelength of λ = 1.54 Å. To obtain a parallel beam, a Goebel mirror was used. The remaining elements of the primary beam optics were a ½ degree divergence slit, a 1.4 mm anti-scatter slit, a 0.04 rad Soller slit, and a 10 mm mask. The intensity of the scattered beam was recorded with a proportional Xe detector equipped with a PPC collimator and a Soller slit of 0.04 rad. The samples were placed on the X-Y-Z-Phi-Chi five-axis universal table enabling precise alignment of the specimens by adjusting their height and tilt angle depending on the plane-parallelism of the tested surfaces. The tests were carried out in the angular range of 2θ = (20–70)° with a 0.05° step and for a 2-s time per step over the entire range of 2θ. The qualitative and quantitative phase analysis of the obtained diffraction patterns was performed using the High Score Plus software provided by the diffractometer manufacturer and the ICDD PDF4 + crystallographic database.

## 3. Results

In Figure 1 the diffraction pattern for the untreated sample is presented. Only the tetragonal phase (Zr-Y-O) were detected.

Figure 2, Figure 3, Figure 4, Figure 5, Figure 6, Figure 7, Figure 8, Figure 9, Figure 10, Figure 11, Figure 12 and Figure 13 present selected diffraction patterns, based on the calculated content of the individual phases of the tested samples.

The presented diffraction patterns show that for each type of treatment, a monoclinic phase appears—a characteristic peak at the 2Theta angle = 28.1°. Depending on the type of abrasive and the processing parameters, the height of the reflections from the individual phase’s changes should be related to the mutual quantitative changes of the monoclinic and tetragonal phases. The calculated contents of the monoclinic phase for the individual types of treatment and the different angles of incidence for the X-ray beam are presented in Table 2 and Table 3

The calculated X-ray penetration depths were as follows:

For the angle of incidence ω = 2°—penetration depth = 1.3 µm
For the angle of incidence ω = 5°—penetration depth = 3.19 µm
For the angle of incidence ω = 10°—penetration depth = 6.35 µm

## 4. Discussion

Diffractometric examinations have shown that the abrasive blasting of zirconium oxide causes a transformation from the tetragonal phase into a monoclinic phase in its surface layer. By analyzing the amount of the monoclinic phase after blasting in relation to the amount of this phase after grinding (presented for the same material in [30]), it can be concluded that abrasive blasting increases the degree of transformation in relation to grinding. This is due to the greater aggressiveness of this treatment related to the greater energy that the abrasive grains have in the air stream in relation to the energy of the grains in the abrasive elements. The same was found in the research by Guazzato and his co-researchers who studied the influence of surface and heat treatment of the material [31]. Using diffraction tests, they showed that sandblasting influences the transformation of the tetragonal phase into a monoclinic phase to a greater extent than grinding, but in both processes this transformation still occurs. On the other hand, He M. and his co-researchers [32], in their work on the effect of sandblasting on the roughness of ZrO_2_ ceramics and the bond strength with veneering ceramics, noticed that sandblasting the material surface only at a pressure of 200 kPa contributes to the transformation of the tetragonal phase into a monoclinic one. Interestingly, the same sample, subjected to the sintering process at a later stage, underwent an inverse transformation—from a monoclinic phase into a tetragonal phase. This fact can be explained by the fact that the temperature at which this process occurs is the temperature in which the tetragonal phase is the stable phase. Therefore, during heating, the transformation of the monoclinic phase into the tetragonal phase takes place.

During their research on the morphology, the structure, and the thermal conductivity of zirconium oxide subjected to the process of sandblasting with aluminium oxide (Al_2_O_3_), Jakovac M. and his co-researchers [33], using SEM tests, noticed that the sample after milling in the CAM/CAM technology contains holes, depressions of the order of 5 µm, which directly affect the quality of the bond with the veneering ceramics, causing it to delaminate and crack with time. However, they noticed that sandblasting the surface with alumina helps to close the cavities after milling the sample, causing it to seal. The milled sample did not contain a monoclinic phase, while the sandblasted samples had a monoclinic phase content of 3–4%. The obtained results concerning the milled samples are analogous to the results presented in this paper. In the sample after the milling process [30], no tetragonal phase was found either. The lack of this phase can also be explained by the fact that the milling process is followed by the sintering process, and at the temperature at which it takes place, the tetragonal phase is the stable phase. Cooling down after the sintering process does not cause the transition to a monoclinic phase (stable at ambient temperature) because the zirconium oxide is stabilized, among others, by yttrium and magnesium oxides, which keep the tetragonal structure at ambient temperatures. There are, however, significant differences in the amount of monoclinic phase occurrences in the sandblasted samples. However, they result from the different geometry of the X-ray beam incidence in the diffraction studies. In the research presented in this paper, the angle of incidence of the beam in relation to the sample surface was very small (2–10°). Therefore, the beam penetrated the sample to a shallow depth. It can be assumed that this was the depth of the impact of the abrasive grains on the material in the sandblasting process. On the other hand, in the research by Jakovac M. and his co-researchers [33], the angle of incidence was greater, so the beam penetrated deeper. The signal was therefore collected both from the area of the impact of the abrasive blasting and from the area not affected by the blasting, where no transformation took place, hence the lower content of the monoclinic phase.

Due to its properties, the monoclinic phase is not desired in prosthetic restorations made of zirconium oxide; however, this cannot be generalized. A certain amount of this phase can have a positive effect on the quality of the restoration. Jakovac M. and co-researchers [33] claim that due to the greater specific volume of the monoclinic phase, during its transformation into the tetragonal phase, the quality of the treated surface improves and the microcracks are closed, and the sandblasting process additionally removes surface impurities of zirconium oxide. Moreover, the increase in volume due to the transformation ahead of the spreading fracture can stop its propagation.

Abrasive blasting is essential for the quality of the bond between the zirconium oxide cup and the veneering ceramic. This treatment is commonly used for other materials used as a cup for prosthetic restorations [28]. As with other materials, it evolves the surface thereby increasing the strength of the connection. It should be noted that in the case of Co-Cr and Ni-Cr alloys, surface oxidation is used to improve the bond, while in the case of zirconium oxide, similarly to titanium, it is impossible. So, all that remains is to increase the surface roughness, and the best results are obtained by sandblasting. Cevik P., Cengiz D., and Malkoc M.A. [26], in their research on the bond strength of veneering porcelain with the zirconium oxide framework after its various surface treatments, pay special attention to the fact that the lack of ZrO_2_ surface treatment results in a reduction in the bond strength with the veneering material. So, to prevent the delamination of the ceramics from the zirconium oxide, its surface should be subjected to a treatment such as grinding or sandblasting. The surface after sandblasting is more advantageous because the traces of processing, unlike grinding, are non-directional and the same strength of the joint can be expected in all directions. The only problem is the selection of appropriate processing parameters (sandblasting pressure, size, and type of abrasive grain), so that the best parameters of surface roughness (responsible for the connection) are obtained, while not retaining too much of the tetragonal phase.

Chintapalli R.K [28] and colleagues in their research on the effect of sandblasting on the surface of zirconium oxide showed that changes in particle size and pressure do not have a large effect on phase changes caused by material erosion. They drew attention to the fact that light sandblasting at a pressure of 2 bar and a grain size of 110 µm can be beneficial for the material because the damage most often only affects the transformed area in which there is a field of compressive stresses. Stronger sandblasting of ZrO_2_ (4 bar, 250 µm), in turn, leads to much greater damage to the material, which cannot be counteracted by the field of compressive stresses. Zhang Y. [25], together with their fellow researchers, subjected sandblasted specimens to cyclic loads on the long-term performance and lifetime of dental ceramics. With the increasing value of the load and over time, the blasted samples lost their properties—the ceramics in the places subjected to sandblasting began to crack. The results of the tests that were carried out indicated that the surface defects formed exceeded any counterbalancing strengthening effect caused by compressive stresses. Additionally, attention was drawn to the fact that grinding and sanding the prosthetic restoration by the dentist while adjusting it in the oral cavity may only aggravate the adverse effect resulting from the material processing. However, prosthetic crowns on a zirconium oxide substructure should be able to withstand chewing forces even up to 400 N in the long term despite any partial material degradation.

Finger C. and other researchers [34] conducted a study on the effect of sandblasting on surface roughness and residual stresses in zirconium oxide. As part of their research, they showed that the highest values for both parameters are obtained when the sandblasting of the surface with aluminum oxide is performed at an angle of 900. It seems that in prosthetic practice, considering the influence of the sandblasting angle does not make sense. Due to the complicated shape of the prosthetic elements, we are not able to maintain a constant angle for different surfaces. Therefore, the most important influence is pressure, grain size, and the type of abrasive.

When we are analyzing the parameters of abrasive blasting, any influence on the transformation of the tetragonal phase into a monoclinic phase should also be considered. As it can be seen from the presented research results, the rate of monoclinic phase transformation in the surface layer of the treated zirconium oxide can even reach 50%. It all depends on the sandblasting pressure, the type of abrasive used, and the grain size of the abrasive. Generally, there is a tendency whereby as the treatment pressure increases, the prevalence of the monoclinic phase increases with the same grain size. Similarly, an increase in the prevalence of the monoclinic phase is observed with an increase in grain size, with the same sandblasting pressure, which in both cases can be explained by an increase in the energy of the incident abrasive particles. Greater pressure leads to greater speed, and therefore greater energy. Larger grains lead to more weight and more energy. The presented research results also showed the influence of the type of abrasive material. There are clearly fewer monoclinic phases in the samples treated with silicon carbide compared to those treated with aluminium oxide with the same parameters. This appears to be due to the properties of silicon carbide. It is a material harder than aluminium oxide and its grains are more sharp-edged. Therefore, cutting is more effective during machining and there is less stress caused by the impact of grains.

The analysis of the content of the individual phases as a function of the depth of the penetration of the X-ray radiation shows that the closer to the surface, the greater the transformation occurs (the greater the share of the monoclinic phase). Unfortunately, based on these studies, it is not possible to unequivocally state at which depth the transformation takes place during abrasive blasting.

During the analysis of the obtained diffraction patterns, the potential for the occurrence of the cubic phase of zirconium oxide was found. This phase is a high-temperature phase and appears only at temperatures above 2370 °C. There are, however, other reports of its presence at ambient temperatures [35,36]. However, an unambiguous determination of its presence in the tested samples is very difficult because the reflections from it lie very close to the reflections from the tetragonal phase and it is impossible to carry out their unequivocal deconvolution.

The following is a summary of the diffraction test results regarding the connection of zirconium oxide cup- dental veneering ceramics. It should be expected that the mechanical treatment (sandblasting and grinding) carried out to develop the surface and increase roughness of ZrO_2_ results in the appearance of an unfavourable monoclinic phase in the surface layers, which may affect the durability of the prosthetic element. As a further consequence, studies of the influence of abrasive blasting parameters on the quality of the bond between the metal cup and veneering ceramics should be carried out, followed by long-term clinical observations of the behavior of the restorations in patients.

## 5. Conclusions and Future Perspectives

Based on presented research, the following conclusions can be drawn.

1. The higher the machining pressure, the greater the prevalence of the monoclinic phase and the deeper the transformation takes place—this is most likely due to the energy carried by the abrasive particles.
2. The larger the grain, the more the monoclinic phase is involved and the deeper the transformation is related to the mass of the particles, and thus the energy they carry.
3. The visible broadening of the reflex characteristic of the tetragonal phase at the 2Theta angle of about 30° may be the result of the appearance of the yttrium-rich regular phase.
4. The closer to the surface, the more the transformation occurs.

Further research should focus, on the one hand, on determining the influence of the degree of the transformation of the tetragonal phase into the monoclinic phase on selected mechanical properties, especially fracture toughness. On the other hand, considering the necessity of veneering the framework with ZrO_2_ ceramics, the surface parameters influencing the quality of the connection should be determined (roughness, free energy of the surface, wettability, and surface topography). The final test should be to determine the strength of the joint and fractographic tests, allowing researchers to determine the course of the cracking, and thus enabling a determination of the weakest link in the joint zirconium oxide framework—veneering ceramics.

## Figures and Tables

**Figure 2:**
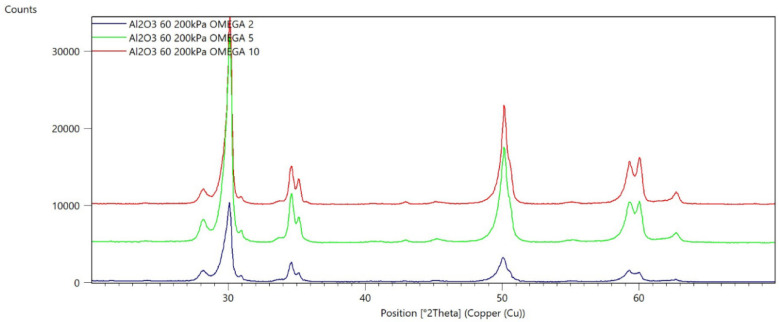
Diffraction patterns of sample A (abrasive—Al_2_O_3_ 60 µm, pressure 200 kPa).

**Figure 3:**
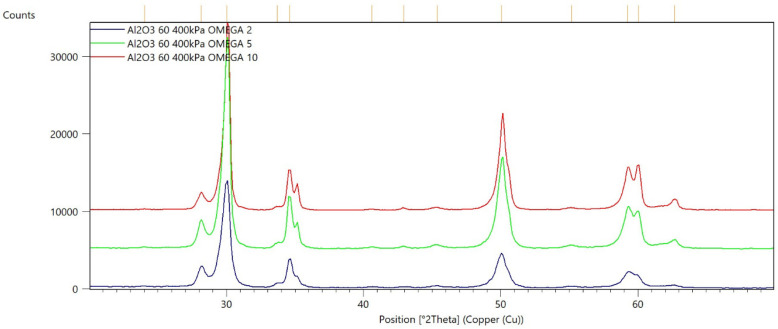
Diffraction patterns of sample B (abrasive—Al_2_O_3_ 60 µm, pressure 400 kPa).

**Figure 4:**
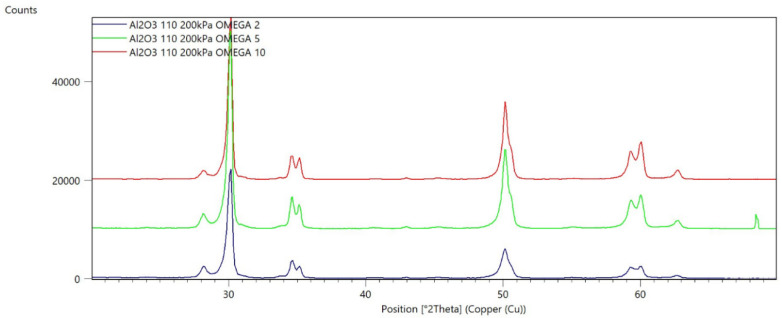
Diffraction patterns of sample C (abrasive—Al_2_O_3_ 110 µm, pressure 200 kPa).

**Figure 5:**
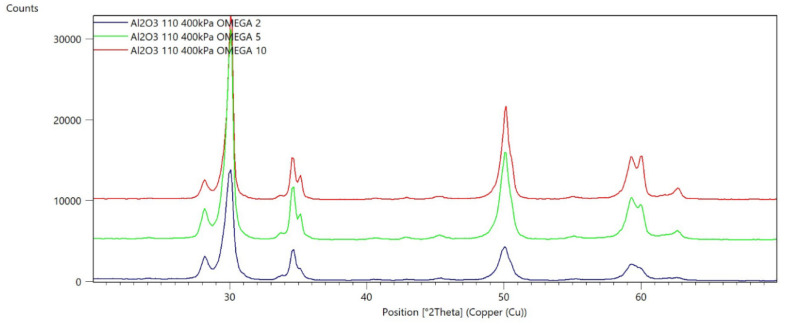
Diffraction patterns of sample D (abrasive—Al_2_O_3_ 110 µm, pressure 400 kPa).

**Figure 6:**
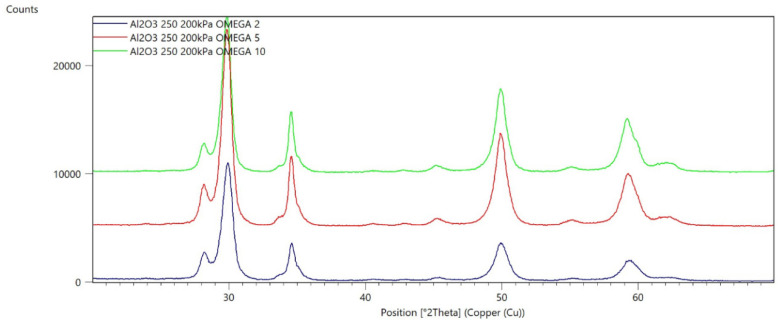
Diffraction patterns of sample E (abrasive—Al_2_O_3_ 250 µm, pressure 200 kPa).

**Figure 7:**
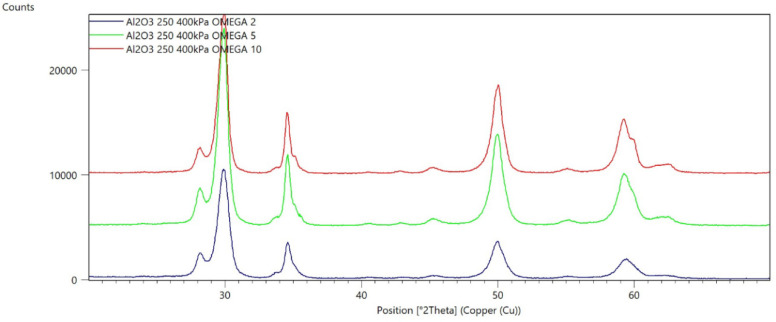
Diffraction patterns of sample F (abrasive—Al_2_O_3_250 µm, pressure 400 kPa).

**Figure 8:**
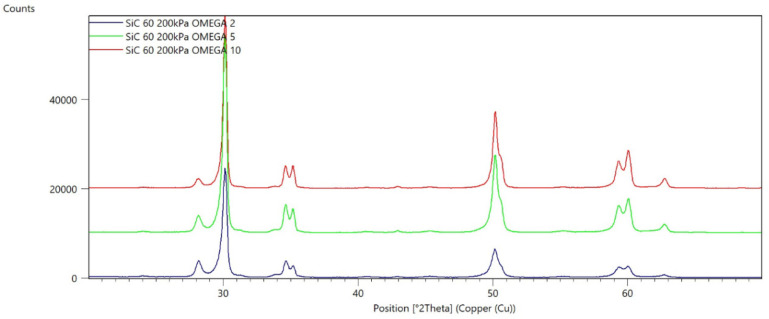
Diffraction patterns of the G sample (abrasive—SiC 60 µm, pressure 200 kPa).

**Figure 9:**
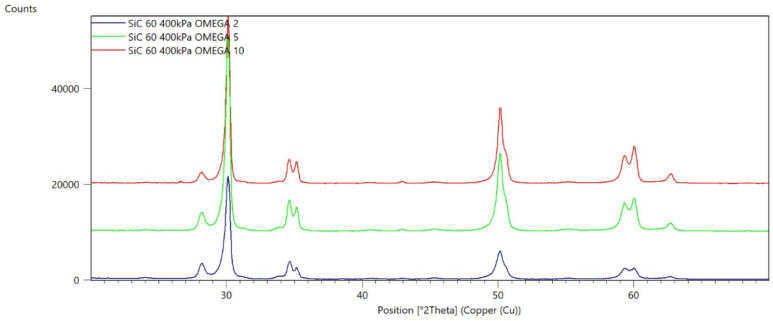
Diffraction patterns of the H sample (abrasive—SiC 60 µm, pressure 400 kPa).

**Figure 10:**
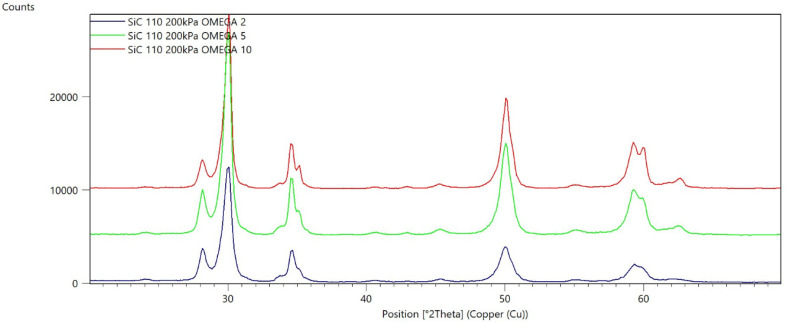
Diffraction patterns of sample I (abrasive—SiC 110 µm, pressure 200 kPa).

**Figure 11:**
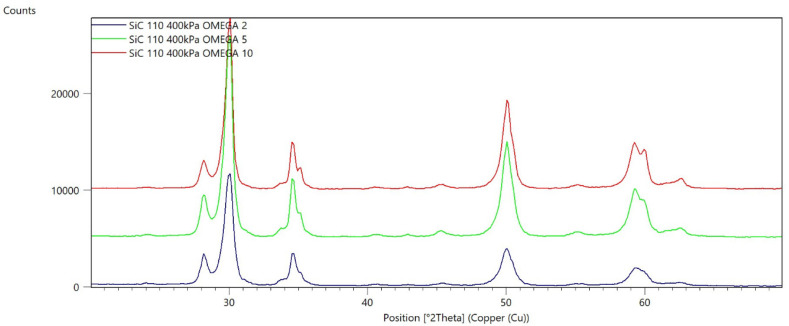
Diffraction patterns of sample J (abrasive—SiC 110 µm, pressure 400 kPa).

**Figure 12:**
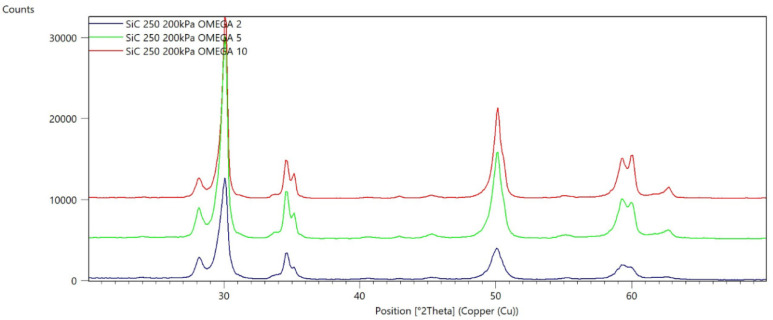
Diffraction patterns of sample K (abrasive—SiC 250 µm, pressure 200 kPa).

**Figure 13:**
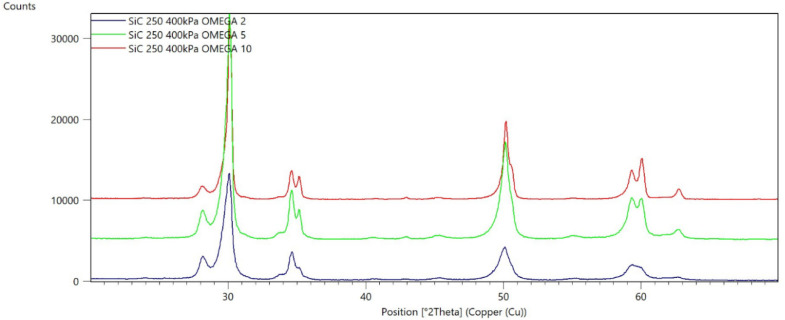
Diffraction patterns of sample L (abrasive—SiC 250 µm, pressure 400 kPa).

**Table 1:** Composition of the tested material (according to the manufacturer’s information).

ZrO_2_ + HfO_2_ + Y_2_O_3_	>99.9
Y_2_O_3_	4.5–5.4
HfO_2_	<5
Al_2_O_3_	<0.5
other oxides	<0.5

**Table 2:** The content of individual phases in the tested Al_2_O_3_ samples.

Type of Abrasive	Pressure	Grain Size [m]	Beam Angle [°]	Phase Content	
	[kPa]			[Weight %]	
				YSZ-T	YSZ-M
Al_2_O_3_	200	60	2	67	33
			5	72	28
			10	76	24
		110	2	74	26
			5	74	26
			10	78	22
		250	2	62	38
			5	56	44
			10	48	52
	400	60	2	62	38
			5	71	29
			10	70	30
		110	2	62	38
			5	68	32
			10	67	33
		250	2	61	39
			5	60	40
			10	59	41

**Table 3:** The content of individual phases in the tested SiC samples.

Type of Abrasive	Pressure	Grain Size [m]	Beam Angle [°]	Phase Content	
	[kPa]			[Weight %]	
				YSZ-T	YSZ-M
SiC	200	60	2	76	24
			5	78	22
			10	81	19
		110	2	61	39
			5	63	37
			10	66	34
		250	2	67	33
			5	69	31
			10	68	32
	400	60	2	75	25
			5	76	24
			10	78	22
		110	2	63	37
			5	63	37
			10	66	34
		250	2	65	35
			5	72	28
			10	74	26

## Data Availability

The data presented in this study are available on request from the corresponding author.
